# Uncovering sociodemographic disparities in temporal trends of osteoarthritis incidence and age-at-diagnosis, 2006–2019

**DOI:** 10.1177/14034948241265427

**Published:** 2024-08-17

**Authors:** Ali Kiadaliri, Martin Englund

**Affiliations:** Faculty of Medicine, Department of Clinical Sciences Lund, Orthopaedics, Clinical Epidemiology Unit, Lund University, Sweden

**Keywords:** Age at diagnosis, intersectional inequality, osteoarthritis, temporal trend, Sweden

## Abstract

**Aim:**

To describe sociodemographic disparities in temporal trends of incidence and age distributions of first registered osteoarthritis (OA) diagnosis in southern Sweden.

**Methods:**

We identified all Skåne residents aged 35+ who had lived in the region at any point during the period 2006–2019 with no previous OA diagnosis (ICD-10 codes M15–M19) for 8 years prior to inclusion in the study (*n* = 849,061). We calculated person-years from inclusion until OA diagnosis, death, emigration, or 31 December 2019, whichever occurred first. Combining sex (female, male), education (low, medium, high) and nativity (Swedish, immigrant), we created a variable with 12 strata. Average annual percent changes in age-standardized incidence rates were estimated using joinpoint regression. Changes in the median age-at-diagnosis (year of diagnosis minus birth year), weighted to the mid-2005 Swedish population, were explored.

**Results:**

Cumulative age-standardized incidence rates ranged from 116 (95% CI: 111, 121) per 10,000 person-years for immigrant males with low education to 205 (95% CI: 200, 210) for immigrant females with medium education. The estimated average annual percent changes (ranging from 3.4% to 6.1%) were generally similar, with slightly greater variations among immigrants than Swedes. The weighted median age-at-diagnosis was higher for Swedes and low educated people. Immigrant females with low education were the only stratum with a reduction (3 years) in the weighted median age-at-diagnosis over time. Sociodemographic patterns in knee OA incidence were different from patterns for hip OA.

**Conclusions:**

There were few sociodemographic disparities in temporal trends of OA incidence and age-at-diagnosis, suggesting persistent sociodemographic disparities in OA burden in southern Sweden.

## Introduction

Osteoarthritis (OA) is a prevalent and debilitating musculoskeletal condition affecting more than 500 million individuals globally [1]. OA is associated with pain, disability, poorer quality of life, and higher healthcare use [2, 3]. Older age, being female, previous joint injury, obesity, high bone mineral density, and family history and genetic predisposition are some of the main risk factors for OA [4]. Due to the aging population, sedentary lifestyles, the lack of a cure, and limited effective symptomatic treatments, the burden of OA has been steadily rising over time [1, 5]. In Sweden, age-standardized hospitalization rates for OA rose by 2.1% annually from 1998 to 2015 [6].

Current evidence suggests that OA prevalence and incidence vary by sociodemographic factors such as sex, race, socioeconomic status, and place of residence [4, 7-11]. For instance, people with lower socioeconomic status have higher OA incidence and prevalence as well as worse clinical outcomes for both knee and hip OA [11]. However, previous studies generally relied on cross-sectional self-reported [4] or register-based data for determining OA incidence [7-9], or have primarily focused on OA prevalence [10]. Consequently, there is a scarcity of evidence regarding sociodemographic variations in temporal trends of OA incidence and the age at which it is diagnosed. Importantly, previous investigations into sociodemographic disparities have often focused on individual sociodemographic factors separately, overlooking the fact that sociodemographic factors are interdependent, mutually constitutive, and reinforce one another. Investigating OA incidence and age-at-diagnosis across different sociodemographic groups will provide crucial insights for developing effective prevention and management strategies. In this study, we aimed to compare temporal trends in OA incidence and age-at-diagnosis across sociodemographic strata defined at the intersections of sex, education, and nativity, using high-quality longitudinal population-based register data from southern Sweden.

## Methods

### Data sources

This is an observational population register-based open cohort study in Skåne, the southernmost region of Sweden. We obtained the individual-level data on age, sex, date of death, residential community, highest educational attainment, and country of birth from the Swedish Population Register and from the Longitudinal Integration Database for Health Insurance and Labour Market Studies (LISA) administered by Statistics Sweden. We retrieved data on all healthcare contacts (primary care, specialized outpatient and inpatient care) in public and private sectors in the region from the Skåne Healthcare Register (SHR). The SHR is an administrative healthcare register holding information on all healthcare consultations in the Skåne region from 1998 onwards [12]. The SHR contains the diagnostic codes from publicly funded clinics according to the International Classification of Diseases 10 (ICD-10) system, assigned by healthcare professionals at the time of the healthcare consultation. It should be noted that while healthcare contacts within private care are recorded in the SHR, the diagnostic codes assigned in private care are not transferred to the SHR. Moreover, healthcare delivered by the municipalities, such as home care, is not registered in the SHR. However, private and municipality care together account for a small proportion of all healthcare in Sweden [12].

### Study population

The study population included all individuals aged 35+ living in the Skåne region at any point between 1 January 2006 and 31 December 2019, who had been living in the region for 8 years prior to inclusion in the study. This means that a person who moved to the region in the year 2010 was included in the study from the year 2018. Applying this 8-year wash-out period to identify incident OA cases was based on a previous study suggesting an average 7.7-year delay between symptom onset and diagnosis of OA [13]. We restricted our sample to persons aged 35+ since OA is uncommon among younger age groups. Moreover, most people have attained their highest level of education by this age. Each individual in the study was followed from their 35^th^ birthday, 8 years after moving to the region, or from 1 January 2006, whichever occurred last, until the first OA registration in the SHR, death, relocation outside of the region, or 31 December 2019, whichever occurred first. Individuals with an OA diagnosis (as the principal or secondary diagnosis) prior to the start of follow-up were excluded. We used both the principal and secondary diagnosis for exclusion to reduce the possibility of including prevalent cases.

### Identification of osteoarthritis

We identified persons with a first OA diagnosis at peripheral joints, registered in the SHR as a principal diagnosis, regardless of the healthcare professional (e.g., doctor, physiotherapist) who registered the diagnosis. The following ICD-10 codes (condition) were used to identify OA diagnosis: M15 (polyarthritis), M16 (hip OA), M17 (knee OA), M18 (OA of the first carpometacarpal joint) and M19 (other OA).

### Sociodemographic strata

We included sex (female, male), educational attainment (low: <10 years, medium: 10–12 years, and high: 13+ years), and nativity (native as those born in Sweden, immigrant as those born abroad) as sociodemographic factors. Educational attainment was measured at the year of inclusion in the study. Using the possible unique combinations of these factors, we created 12 sociodemographic strata: 12 = 2 × 3 × 2.

### Data analysis

For each sociodemographic stratum, we calculated the annual incidence rates as the number of new cases of OA in each calendar year divided by the number of person-years of follow-up in that year. We then estimated age-standardized incidence rates (ASIRs) by means of direct standardization, using Sweden’s population in the year 2005 as standard. We also estimated age-specific incidence rates and the distribution of age-at-diagnosis for two 3-year periods: 2006–2008 and 2017–2019.

We performed joinpoint regression to investigate temporal trends in ASIRs for each sociodemographic stratum using the Joinpoint Regression Program, version 5.0.2 (https://surveillance.cancer.gov/joinpoint/). Given 14 data points, 2006–2019, a maximum of two joinpoints and three trend segments were allowed. We set a minimum number of three observations per segment. The optimal number of joinpoints (0–2) was selected based on data-driven weighted Bayesian information criteria. After selecting the optimal number of joinpoints, the program estimates an annual percentage change for each segment. To provide a summary measure of the trend for the whole period, the average annual percent change (AAPC) was calculated as the weighted average of annual percentage changes. Furthermore, we employed pairwise comparisons to test the parallelism of temporal trends between sociodemographic strata. The distributions of age-at-diagnosis, weighted to the mid-2005 Swedish population, for each stratum for the periods 2006–2008 and 2017–2019 were visualized using kernel density plots, and quantile regression was used to compare the median age-at-diagnosis across sociodemographic strata and between periods, that is, 2006–2008 versus 2017–2019. Data visualization and quantile regression were conducted in Stata 18.

### Subgroup analyses

To account for potential differences in the sociodemographic patterns of knee and hip OA as the two most common types, we conducted separate analyses for hip (ICD-10 code: M16) and knee (ICD-10 code: M17) OA. For instance, while there were racial differences in hip OA incidence between African Americans and Whites, no such differences were observed for knee OA incidence [11].

In a second subgroup analysis, we only included doctor-diagnosed OA and thus excluded diagnoses registered by other health professionals. This subgroup analysis was conducted because during the study period an important structural change in the SHR took place, in which health professionals other than physicians were allowed to register a diagnosis [12].

### Sensitivity analysis

In our main analysis, we excluded those with missing information on educational attainment. To account for the fact that educational attainment is more likely to be missing for older individuals who were born abroad, in a sensitivity analysis we assumed that people missing this had a low level of educational attainment, and explored the effects on our main findings. Of note, the mean age (63.6 years) of those with missing information on education was closer to those with a low (62.3 years) than those with a medium (50.4 years) or high (47.1 years) level of educational attainment in our cohort, rendering our assumption plausible. This sensitivity analysis should be considered as a worst-case scenario.

### Ethics

This study received ethical approval from the Lund University Ethical Review Committee (Dnr 2011-432 and 2014-276) and was performed in accordance with the Declaration of Helsinki.

## Results

After excluding 14,994 (1.8%) individuals with missing information on educational attainment, a total of 834,067 unique individuals, 50.8% female, were included and followed for 7,990,444 person-years. Excluded individuals were, on average, older than those included (63.6 versus 52.2 years) and were predominantly immigrants (78.3%).

**Figure 1.**
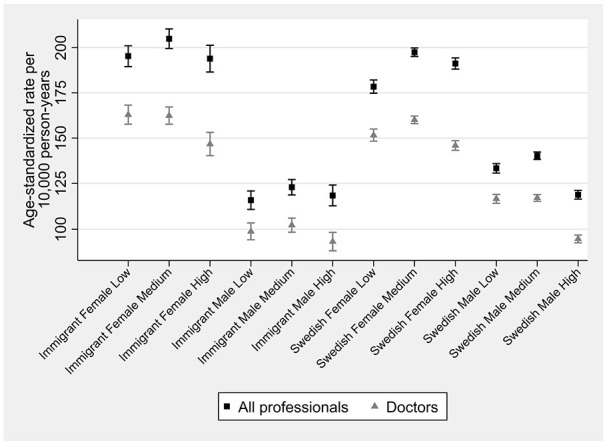
Cumulative age-standardized osteoarthritis incidence rates by sociodemographic strata, 2006–2019.

During follow-up, 125,798 persons (15.1%) had a first registered OA diagnosis, corresponding to a cumulative ASIR of 158 (95% CI: 157, 159) per 10,000 person-years (Table A1 in Supplemental material). The cumulative ASIRs ranged from 116 (95% CI: 111, 121) for immigrant males with low education to 205 (95% CI: 200, 210) for immigrant females with medium education (Figure 1). Females had higher ASIRs than males regardless of educational attainment and nativity. Those with medium education tended to have higher ASIRs than other educational groups. While among females, ASIRs were higher for immigrants than Swedes, the opposite was the case among males, and these disparities declined with increasing education in both sexes. The weighted median age-at-diagnosis was 61 years, ranging from 57 years in immigrant females with medium/high education and immigrant males with medium education to 69 years in Swedish females with low education. The weighted median age-at-diagnosis tended to be higher for Swedish and low educated people (Table A2 in Supplemental material).

### Temporal trends

**Figure 2.**
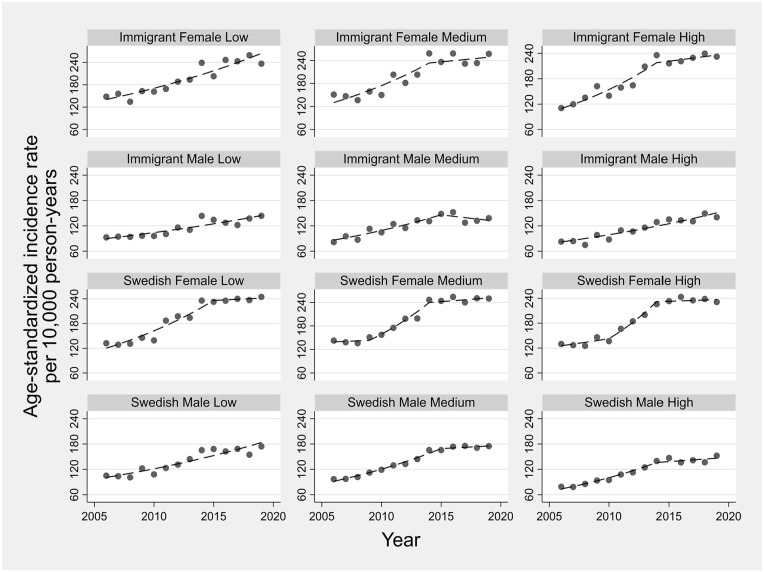
Temporal trends in age-standardized osteoarthritis incidence rates across sociodemographic strata defined by nativity, sex and education. Symbols display observed values and lines display modeled values using joinpoint regression.

**Figure 3.**
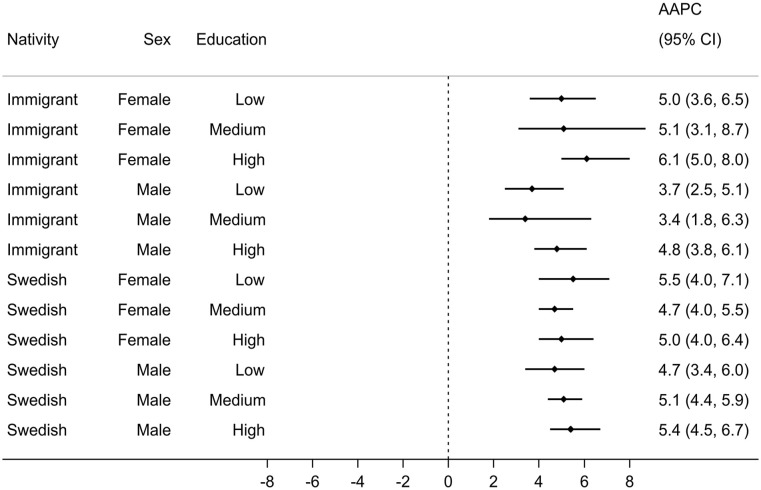
Average annual percent change (AAPC) in age-standardized osteoarthritis incidence rates across sociodemographic strata, 2006–2019.

Annual ASIRs for sociodemographic strata are displayed in Figure 2. In all strata, ASIRs rose over time with most strata experiencing changes (joinpoint) in their temporal trends, with generally increasing trends during the earlier years and stable trends in more recent years. The AAPC ranged from 3.4% (95% CI: 1.8, 6.3) for immigrant males with medium education to 6.1% (95% CI: 5.0, 8.0) for immigrant females with high education (Figure 3). The estimated AAPCs were generally similar, with slightly greater variation among immigrants than Swedish persons. Pairwise comparisons also suggested little disparity in the estimated AAPCs, with only 2 out of 66 possible comparisons showing statistically conclusive differences (*p*-value <0.05) for immigrant males with low education versus immigrant females with high education and Swedish males with high education. Moreover, the parallel trend assumption was rejected in only 15 out of 66 pairwise comparisons, with immigrant males with low and medium education appearing in 11 of these 15 pairs. Most age groups observed increased incidence rates between the periods 2006–2008 and 2017–2019, with more profound rises in females than males (Figure A1 in Supplemental material).

**Figure 4.**
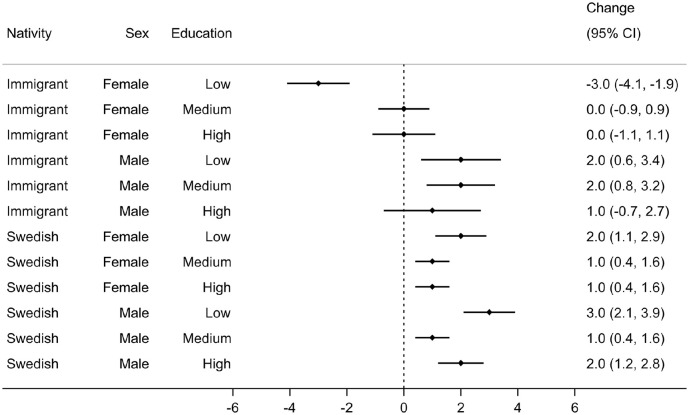
Change (95% CI) in the weighted median age-at-diagnosis between 2006–2008 and 2017–2019 across sociodemographic strata.

The distributions of the weighted age-at-diagnosis generally suggested a shift toward older ages for all sociodemographic strata over time, except for immigrant females with low education (Figure A2 in Supplemental material). The changes in the weighted median age-at-diagnosis ranged from –3 (95% CI: –4.1, –1.9,) years, that is, a 3-year decrease, among immigrant females with low education to +3 (95% CI: 2.1, 3.9) years, that is, a 3-year increase, among Swedish males with low education between 2006–2008 and 2017–2019 (Figure 4).

### Subgroup analyses

Our subgroup analysis suggested different sociodemographic patterns for knee OA compared with hip OA. While the highest cumulative ASIRs for knee OA were reported among immigrant females with low education, the highest ASIRs for hip OA were seen among Swedish females with high education (Figure A3 in Supplemental material). Persons with high education had lower cumulative ASIRs for knee OA than other educational groups, whereas the opposite was seen for hip OA. For knee OA, all sociodemographic strata observed rising trends in ASIRs with the AAPC ranging from 2.3% (95% CI: 0.4, 4.4) in immigrant males with low education to 7.1% (95% CI: 5.0, 10.0) in immigrant females with high education (Figure A4 in Supplemental material). Notably, all immigrant strata experienced continuously rising trends in ASIRs of knee OA with no joinpoint (Figure A5 in Supplemental material). In general, there were rising trends for ASIRs of hip OA, even though this was less pronounced among immigrants (Figure A6 in Supplemental material). AAPC ranged from 0.8% (95% CI: –2.3, 4.1) in immigrant females with low education to 5.5% (95% CI: 3.3, 7.2) in Swedish females with medium education. No strata except Swedish males with high education observed joinpoints in ASIRs of hip OA over the study period (Figure A7 in Supplemental material). The AAPC magnitude was generally greater for knee than hip OA. The distributions of weighted age-at-diagnosis suggested an upward shift for knee OA for most strata, whereas this was less evident for hip OA (Figure A8 in Supplemental material). For all sociodemographic strata, the weighted median age-at-diagnosis was higher for hip than knee OA, with the greatest difference being 6 years among immigrant females with low education (Table A3 in Supplemental material).

**Figure 5.**
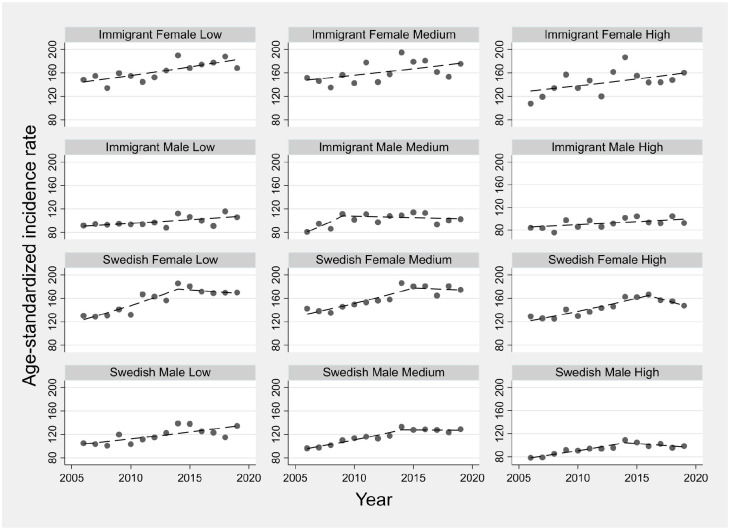
Temporal trends in age-standardized doctor-diagnosed osteoarthritis incidence rates across sociodemographic strata. Symbols display observed values and lines display modeled values using joinpoint regression.

Including only doctor-diagnosed OA gave 104,351 incident cases, corresponding to 12.5% of the sample, and a cumulative ASIR of 129 (95% CI: 128, 130) per 10,000 person-years (Table A4 in Supplemental material). Sociodemographic patterns were similar to those diagnosed by all healthcare professionals (Figure 1). Whereas most immigrant strata experienced a persistent rising trend (i.e., no joinpoint), most Swedish strata had experienced a stable trend during recent years (Figure 5). The magnitude of the AAPCs dropped substantially compared with the main analysis (Figure A9 in Supplemental material), but aligned with the main analysis, the magnitude of the AAPCs were similar across sociodemographic strata. For most sociodemographic strata, the weighted median age-at-diagnosis was identical to the main analysis. The sociodemographic patterns of change in the weighted median age-at-diagnosis were also similar to the main analysis, with immigrant females with low education being the only group experiencing a decline in the median value (Figure A10 in Supplemental material).

### Sensitivity analysis

Assigning those with missing information on education to the low education group yielded almost identical results to our main analysis (Table A5 in Supplemental material).

## Discussion

To the best of the authors’ knowledge, this is the first time that intersectional disparities in the temporal trends of OA incidence and age-at-diagnosis using population register-based data have been explored. Our results suggested similar rising trends in age-standardized OA incidence across sociodemographic strata defined by sex, education, and nativity. Moreover, most strata experienced an upward shift in the weighted median age-at-diagnosis.

The higher OA incidences for females and those with medium education found are in line with our previous study exploring intersectional inequalities in OA incidence among persons aged 40–65 in the Skåne region [8]. The increasing trends in OA incidence found in our study are consistent with global trends [1, 5] and country-specific trends [14-17], even though both rising and declining trends have been documented in the UK [18-20]. In general, previous studies have investigated temporal trends by sex, and reported similar trends for males and females, which is consistent with our findings. Similar temporal trends in OA incidence across sociodemographic strata in our study imply that there were few sociodemographic variations in the mechanisms underlying rising OA incidence, including epidemiological and administrative changes such as coding practices during the study period. This also suggests that sociodemographic disparities in OA incidence in the region were persistent over the study period, which is consistent with previous findings in the UK [9].

We observed a tendency toward a younger weighted median age-at-diagnosis among immigrants and those with high education. A previous study in the UK [21] reported a younger median age-at-diagnosis in the most deprived compared with the least deprived communities, where a higher proportion of immigrants and low educated people reside in the former communities (https://www.ethnicity-facts-figures.service.gov.uk/uk-population-by-ethnicity/demographics/people-living-in-deprived-neighbourhoods/latest/). However, given the difference in area-level socioeconomic measures used in the UK study compared to the individual-level measure in ours, these studies are not directly comparable. Moreover, it has been suggested that area-level socioeconomic measures have limitations as proxies for individual-level socioeconomic status, may be vulnerable to ecological fallacy and, therefore, should be used with caution [22, 23]. Although the weighted median age-at-diagnosis was stable in the whole sample between the periods 2006–2008 and 2017–2019, most sociodemographic strata observed an upward shift in the weighted median age-at-diagnosis. Sociodemographic disparities in age-at-diagnosis and its temporal changes might reflect sociodemographic differences in the epidemiological and clinical profiles of OA, and the prevalence of OA risk factors, including genetic predisposition, access to healthcare, health-seeking behaviors, and coding practices. For instance, immigrant workers are overrepresented among manual unskilled jobs [24], which can raise the risk of OA incidence at a younger age. Younger age-at-diagnosis among immigrants might also suggest the “salmon bias,” which states that immigrants with poor health are more likely to return to their country of origin [25]. Accordingly, if elderly immigrants return to their country of origin prior to an OA diagnosis, this could translate into a younger age-at-diagnosis for immigrants.

Using high-quality individual-level register data spanning 20 years on the whole Skåne population is the main strength of the current study. However, several limitations should be acknowledged. As an administrative data source, the SHR is prone to misdiagnosis and coding errors [12], and possible sociodemographic variations in these errors could have biased our estimates. Although knee OA diagnoses in the SHR have been previously validated [26], other OA diagnoses have not. Since diagnostic codes from private caregivers are not recorded in the SHR, our results are likely to underestimate OA incidence for those sociodemographic strata who mainly receive their care from private caregivers. However, given the small share of private care in Sweden, we do not expect that his will have had a significant effect on our estimates. Using a regional register implies that receiving an OA diagnosis in another region of Sweden or country of origin (for immigrants) cannot be ruled out. However, given the inclusion criterion of the study, living for 8 years in the region, this is very unlikely to have had an influence on our estimates. While applying an 8-year wash-out period makes it unlikely, the inclusion of prevalent cases cannot be ruled out. This is a descriptive epidemiological study, and all given explanations for sociodemographic disparities in OA incidence trends are speculative.

## Conclusion

We observed similar rising trends in OA incidence across sociodemographic strata defined by sex, education, and nativity. We also observed a general upward shift in age-at-diagnosis across sociodemographic strata over time. These results suggest persistent sociodemographic disparities in OA incidence, which calls for improving OA prevention and management strategies.

## Supplemental Material

sj-docx-1-sjp-10.1177_14034948241265427 – Supplemental material for Uncovering sociodemographic disparities in temporal trends of osteoarthritis incidence and age-at-diagnosis, 2006–2019 Supplemental material, sj-docx-1-sjp-10.1177_14034948241265427 for Uncovering sociodemographic disparities in temporal trends of osteoarthritis incidence and age-at-diagnosis, 2006–2019 by Ali Kiadaliri and Martin Englund in Scandinavian Journal of Public Health
