# Supplementary material for: Uncovering sociodemographic disparities in temporal trends of osteoarthritis incidence and age-at-diagnosis, 2006–2019
Source: Scand J Public Health. 2024 Aug 17;53(7):747–55. doi: 10.1177/14034948241265427 (PMC12598052; doi:10.1177/14034948241265427)

**Table A1. The number of osteoarthritis incidence, crude rate, and median age-at-diagnosis during 2006-2019, by sociodemographic strata.**

|  | N | # of incidence diagnosis | Crude rate per 10,000 person-years | Weighted median (IQR) age-at-diagnosis |
| --- | --- | --- | --- | --- |
| Immigrant Female Low | 24204 | 4358 | 201 (195, 207) | 59 (53-67) |
| Immigrant Female Medium | 42041 | 6654 | 169 (165, 174) | 57 (51-63) |
| Immigrant Female High | 40688 | 4274 | 130 (126, 134) | 57 (51-63) |
| Immigrant Male Low | 21380 | 2115 | 113 (109, 118) | 60 (52-67) |
| Immigrant Male Medium | 46727 | 4378 | 102 (99, 105) | 57 (50-63) |
| Immigrant Male High | 36769 | 2529 | 86 (82, 89) | 58 (50-65) |
| Swedish Female Low | 77721 | 16854 | 243 (239, 246) | 69 (61-77) |
| Swedish Female Medium | 130451 | 26819 | 199 (197, 202) | 60 (54-68) |
| Swedish Female High | 108468 | 16722 | 159 (157, 161) | 61 (54-68) |
| Swedish Male Low | 77815 | 12783 | 170 (167, 173) | 65 (58-73) |
| Swedish Male Medium | 133960 | 18467 | 134 (132, 136) | 60 (52-67) |
| Swedish Male High | 93843 | 9845 | 107 (105, 109) | 62 (54-69) |
| Total | 834067 | 125798 | 157 (157, 158) | 61 (54-69) |

IQR: Interquartile range.

**Table A2. Difference (95% CI) in the weighted median age of osteoarthritis diagnosis across sociodemographic strata, 2006-2019.**

| Strata | Any | Knee | Hip |
| --- | --- | --- | --- |
| Immigrant Female Low | Ref | Ref | Ref |
| Immigrant Female Medium | -2 (-2.6, -1.4) | -2 (-2.6, -1.4) | -6 (-7.7, -4.3) |
| Immigrant Female High | -2 (-2.6, -1.4) | -2 (-2.6, -1.4) | -6 (-7.8, -4.2) |
| Immigrant Male Low | 1 (0.2, 1.8) | 1 (0.2, 1.8) | -3 (-5.1, -0.9) |
| Immigrant Male Medium | -2 (-2.6, -1.4) | -2 (-3.0, -1.0) | -6 (-7.8, -4.2) |
| Immigrant Male High | -1 (-1.7, -0.3) | -1 (-2.3, 0.3) | -5 (-7.0, -3.0) |
| Swedish Female Low | 10 (9.7, 10.3) | 10 (9.3, 10.7) | 7 (5.6, 8.4) |
| Swedish Female Medium | 1 (0.7, 1.3) | 1 (0.5, 1.5) | -1 (-2.4, 0.4) |
| Swedish Female High | 2 (1.7, 2.3) | 2 (1.5, 2.5) | -2 (-3.4, -0.6) |
| Swedish Male Low | 6 (5.7, 6.3) | 5 (4.5, 5.5) | 3 (1.5, 4.5) |
| Swedish Male Medium | 1 (0.7, 1.3) | 0 (-0.5, 0.5) | -3 (-4.4, -1.6) |
| Swedish Male High | 3 (2.5, 3.5) | 2 (1.3, 2.7) | -2 (-3.5, -0.5) |

**Table A3. The number of knee/hip osteoarthritis incidence, crude rate and median age-at-diagnosis during 2006-2019, by sociodemographic strata.**

|  | N | # of incidence diagnosis | | Crude rate per 10,000 person-years | | Weighted median (IQR) age-at-diagnosis | |
| --- | --- | --- | --- | --- | --- | --- | --- |
|  |  | Knee | Hip | Knee | Hip | Knee | Hip |
| Immigrant Female Low | 24204 | 2032 | 509 | 94 (90, 98) | 23 (22, 26) | 59 (53-67) | 65 (58-73) |
| Immigrant Female Medium | 42041 | 2702 | 767 | 69 (66, 71) | 20 (18, 21) | 57 (51-63) | 59 (52-66) |
| Immigrant Female High | 40688 | 1686 | 578 | 51 (49, 54) | 18 (16, 19) | 57 (52-63) | 59 (52-66) |
| Immigrant Male Low | 21380 | 963 | 366 | 52 (48, 55) | 20 (18, 22) | 60 (52-66) | 62 (56-71) |
| Immigrant Male Medium | 46727 | 2012 | 664 | 47 (45, 49) | 15 (14, 17) | 57 (50-63) | 59 (51-68) |
| Immigrant Male High | 36769 | 1114 | 415 | 38 (36, 40) | 14 (13, 15) | 58 (51-65) | 60 (51-68) |
| Swedish Female Low | 77721 | 6815 | 3227 | 98 (96, 101) | 46 (45, 48) | 69 (61-77) | 72 (65-79) |
| Swedish Female Medium | 130451 | 10628 | 4383 | 79 (77, 80) | 33 (32, 34) | 60 (54-68) | 64 (56-72) |
| Swedish Female High | 108468 | 6655 | 2976 | 63 (62, 65) | 28 (27, 29) | 61 (54-67) | 63 (56-70) |
| Swedish Male Low | 77815 | 5889 | 2853 | 78 (76, 80) | 38 (37, 39) | 64 (58-73) | 68 (61-75) |
| Swedish Male Medium | 133960 | 8590 | 3548 | 62 (61, 64) | 26 (25, 27) | 59 (52-67) | 62 (55-71) |
| Swedish Male High | 93843 | 4538 | 2141 | 49 (48, 51) | 23 (22, 24) | 61 (54-68) | 63 (56-70) |
| Total | 834067 | 53624 | 22427 | 67 (67, 68) | 28 (28, 28) | 61 (54-68) | 64 (57-72) |

IQR: Interquartile range.

**Table A4. The number of doctor-diagnosed osteoarthritis incidence, crude rate and median age-at-diagnosis during 2006-2019, by sociodemographic strata.**

|  | N | # of incidence diagnosis | Crude rate per 10,000 person-years | Weighted median (IQR) age-at-diagnosis |
| --- | --- | --- | --- | --- |
| Immigrant Female Low | 24204 | 3695 | 168 (163, 174) | 60 (53, 68) |
| Immigrant Female Medium | 42041 | 5330 | 134 (130, 138) | 57 (51, 63) |
| Immigrant Female High | 40688 | 3258 | 98 (95, 102) | 57 (51, 63) |
| Immigrant Male Low | 21380 | 1818 | 97 (92, 101) | 60 (52, 67) |
| Immigrant Male Medium | 46727 | 3666 | 85 (82, 87) | 57 (49, 63) |
| Immigrant Male High | 36769 | 2022 | 68 (65, 71) | 58 (50, 65) |
| Swedish Female Low | 77721 | 14723 | 209 (206, 212) | 69 (61, 77) |
| Swedish Female Medium | 130451 | 22130 | 162 (160, 164) | 61 (54, 68) |
| Swedish Female High | 108468 | 12984 | 122 (120, 124) | 61 (54, 68) |
| Swedish Male Low | 77815 | 11235 | 148 (146, 151) | 65 (58, 73) |
| Swedish Male Medium | 133960 | 15558 | 112 (110, 114) | 60 (52, 67) |
| Swedish Male High | 93843 | 7932 | 85 (84, 87) | 61 (54, 69) |
| Total | 834067 | 104351 | 129 (128, 130) | 61 (54, 69) |

IQR: Interquartile range.

**Table A5. The results of sensitivity analysis assigning those with missing education to low education group.**

|  | N | # of incidence diagnosis | Crude rate per 10,000 person-years | Weighted median (IQR) age-at-diagnosis | Change in Weighted median age-at-diagnosis | AAPC (95% CI) |
| --- | --- | --- | --- | --- | --- | --- |
| Immigrant Female Low | 29432 | 4958 | 198 (193, 204) | 60 (53-68) | -4.0 (-5.1, -2.9) | 4.9 (3.8, 6.2) |
| Immigrant Male Low | 26836 | 2319 | 108 (104, 113) | 60 (52-67) | 2.0 (0.3, 3.7) | 3.5 (2.2, 5.0) |
| Swedish Female Low | 80384 | 17039 | 241 (238, 245) | 69 (61-77) | 2.0 (1.1, 2.9) | 5.4 (4.0, 7.0) |
| Swedish Male Low | 79462 | 12898 | 169 (167, 172) | 65 (58-73) | 3.0 (2.1, 3.9) | 4.7 (3.3, 6.0) |
| Total | 849061 | 126902 | 157 (156, 158) | 61 (54-69) | 0.0 (-0.3, 0.3) | - |

IQR: Interquartile range, AAPC: Average annual percent change.

**Figure A1. Age-specific osteoarthritis incidence rates per 10,000 person-years for 2006-2008 (grey lines with circles) and 2017-2019 (black dashed lines with squares) across sociodemographic strata.**


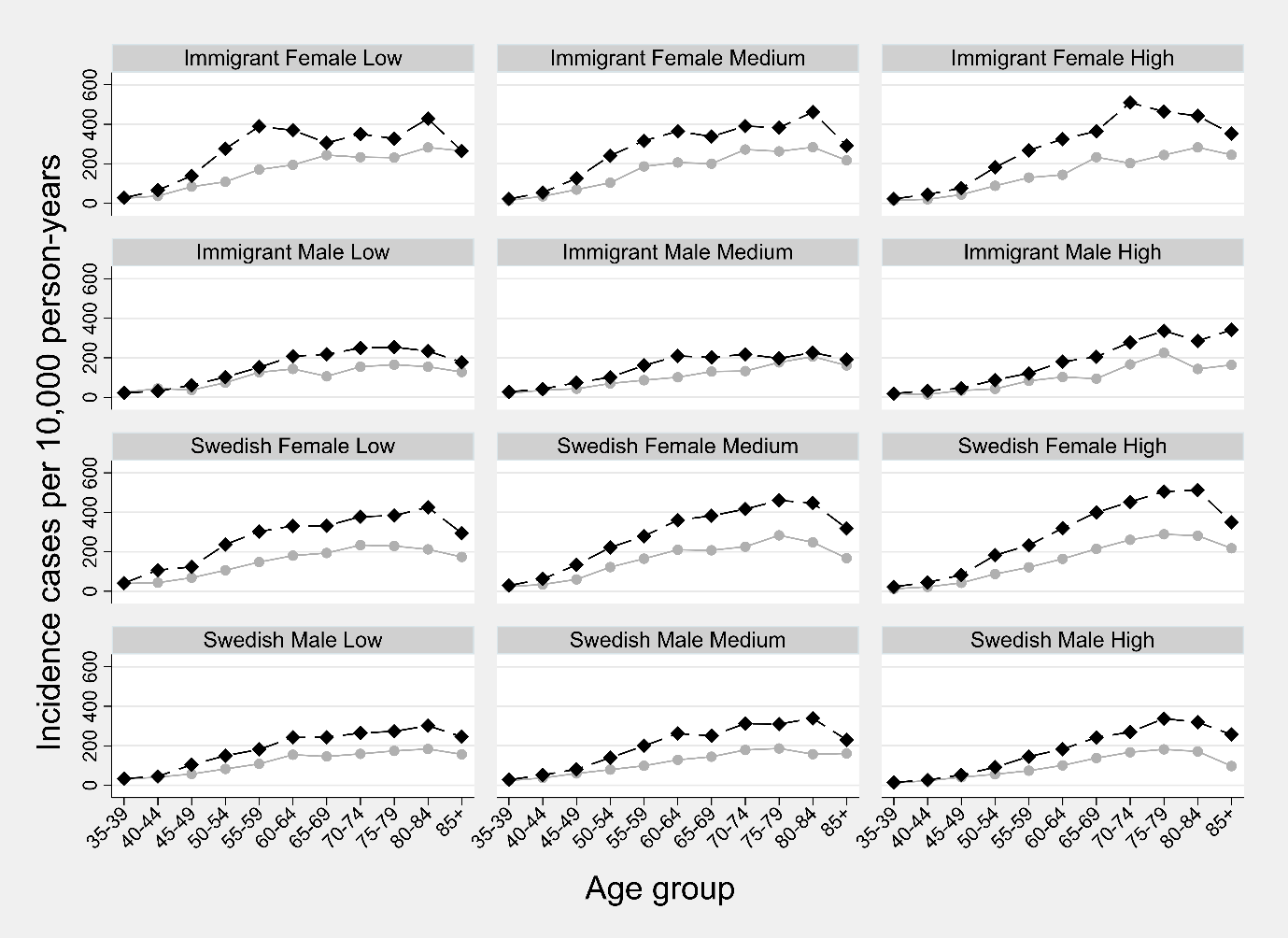


**Figure A2. Changes in the distribution of weighted age at osteoarthritis diagnosis between 2006-2008 (solid line) and 2017-2019 (dashed line) across sociodemographic strata.**


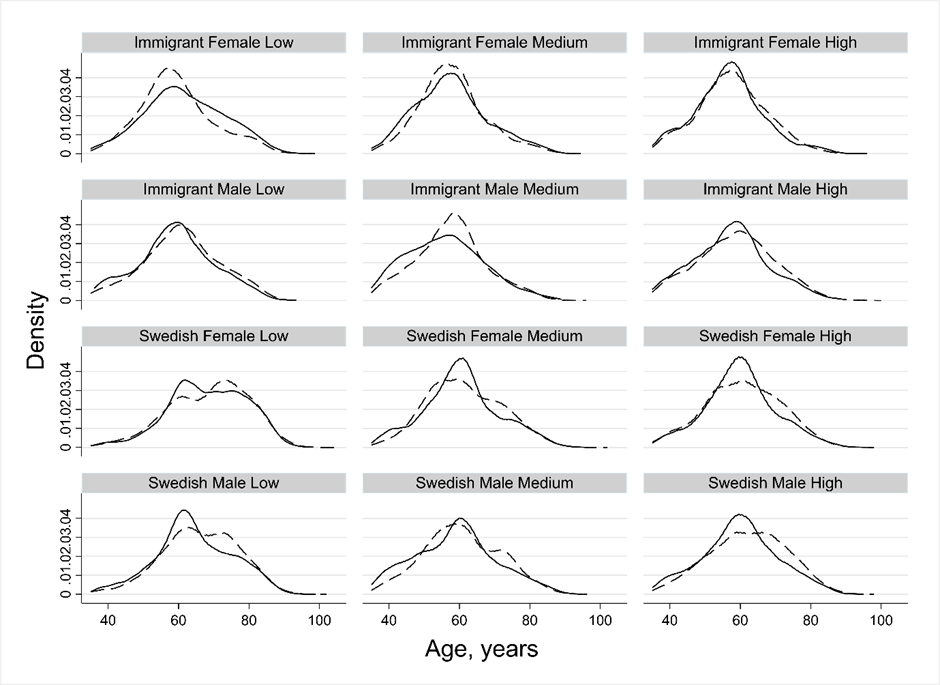


**Figure A3. Cumulative age-standardized knee and hip osteoarthritis incidence rates by sociodemographic strata, 2006-2019.**


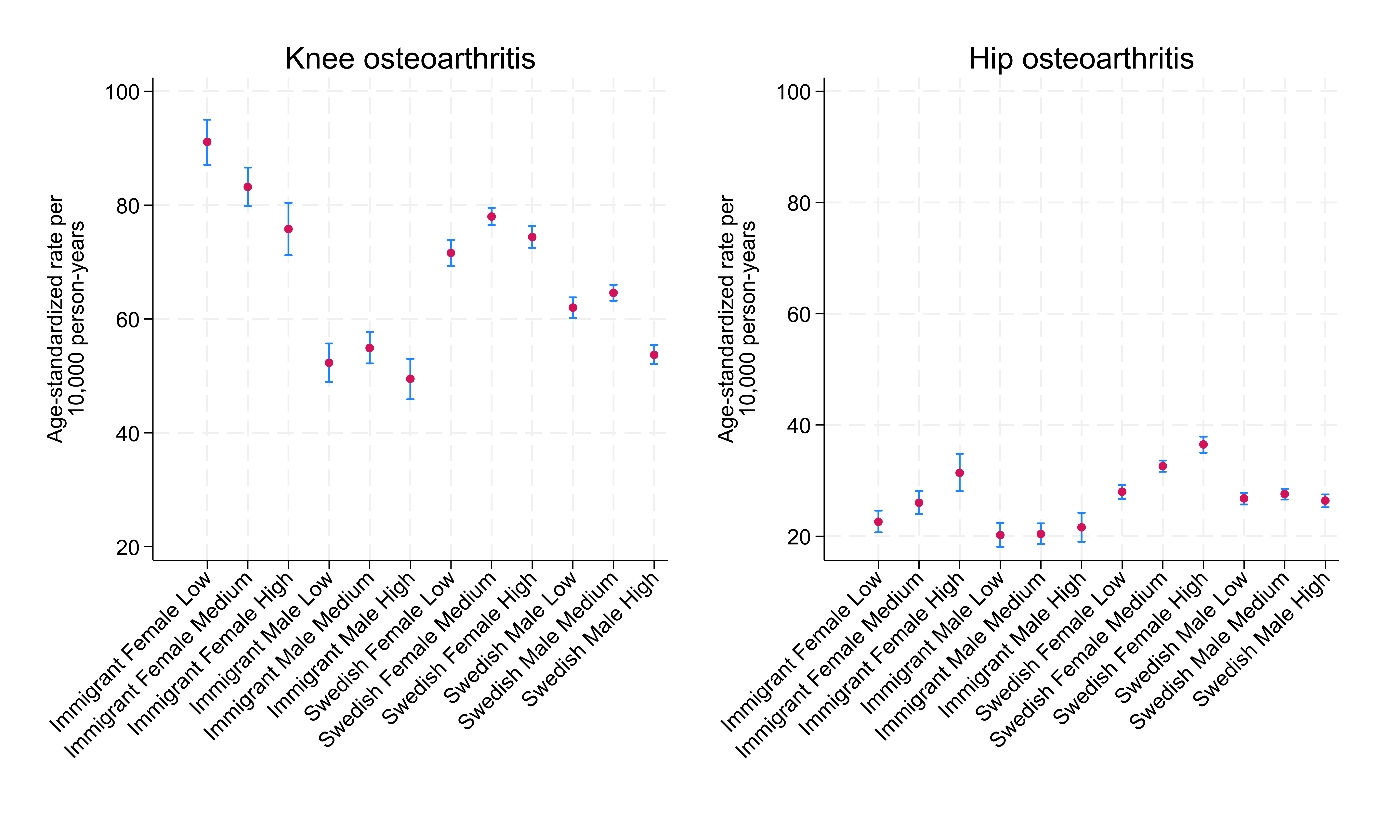


**Figure A4. Average annual percent change (AAPC) in age-standardized knee osteoarthritis incidence rates across sociodemographic strata during 2006-2019.**

**
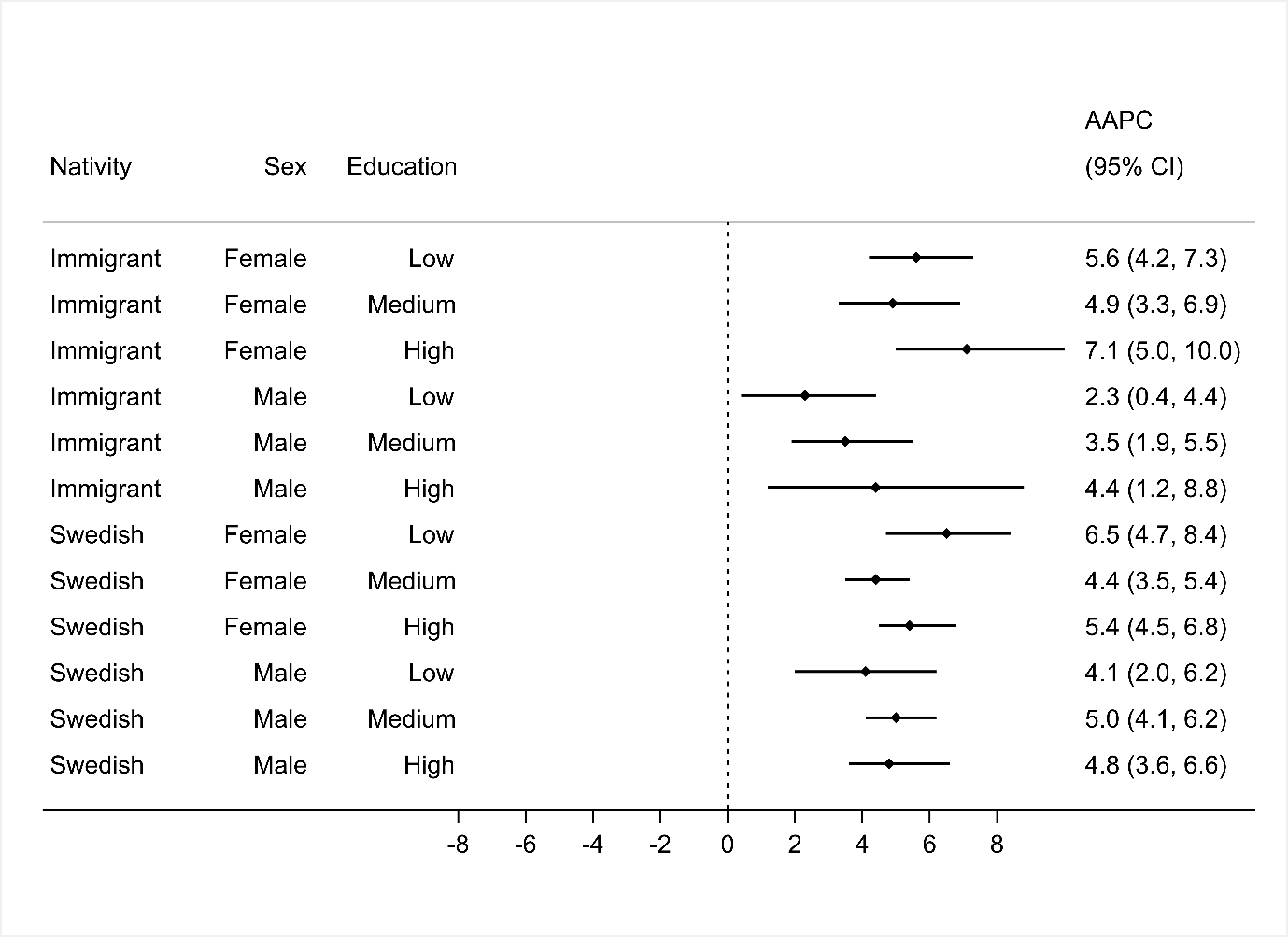
**

**Figure A5. Temporal trends in age-standardized knee osteoarthritis incidence rates across sociodemographic strata. Symbols display observed values and lines display modelled values using joinpoint regression.**


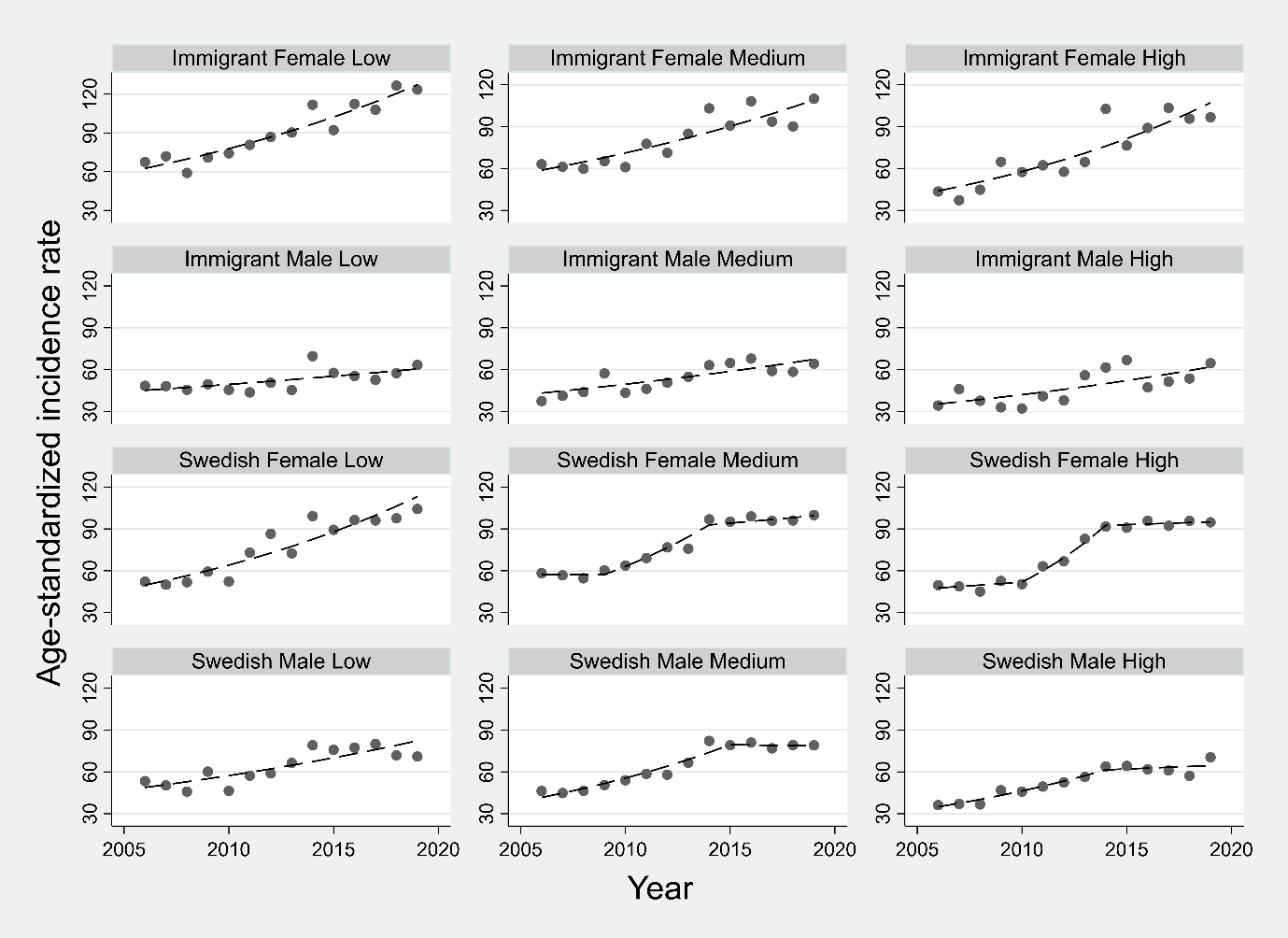


**Figure A6. Average annual percent change (AAPC) in age-standardized hip osteoarthritis incidence rates across sociodemographic strata during 2006-2019.**

**
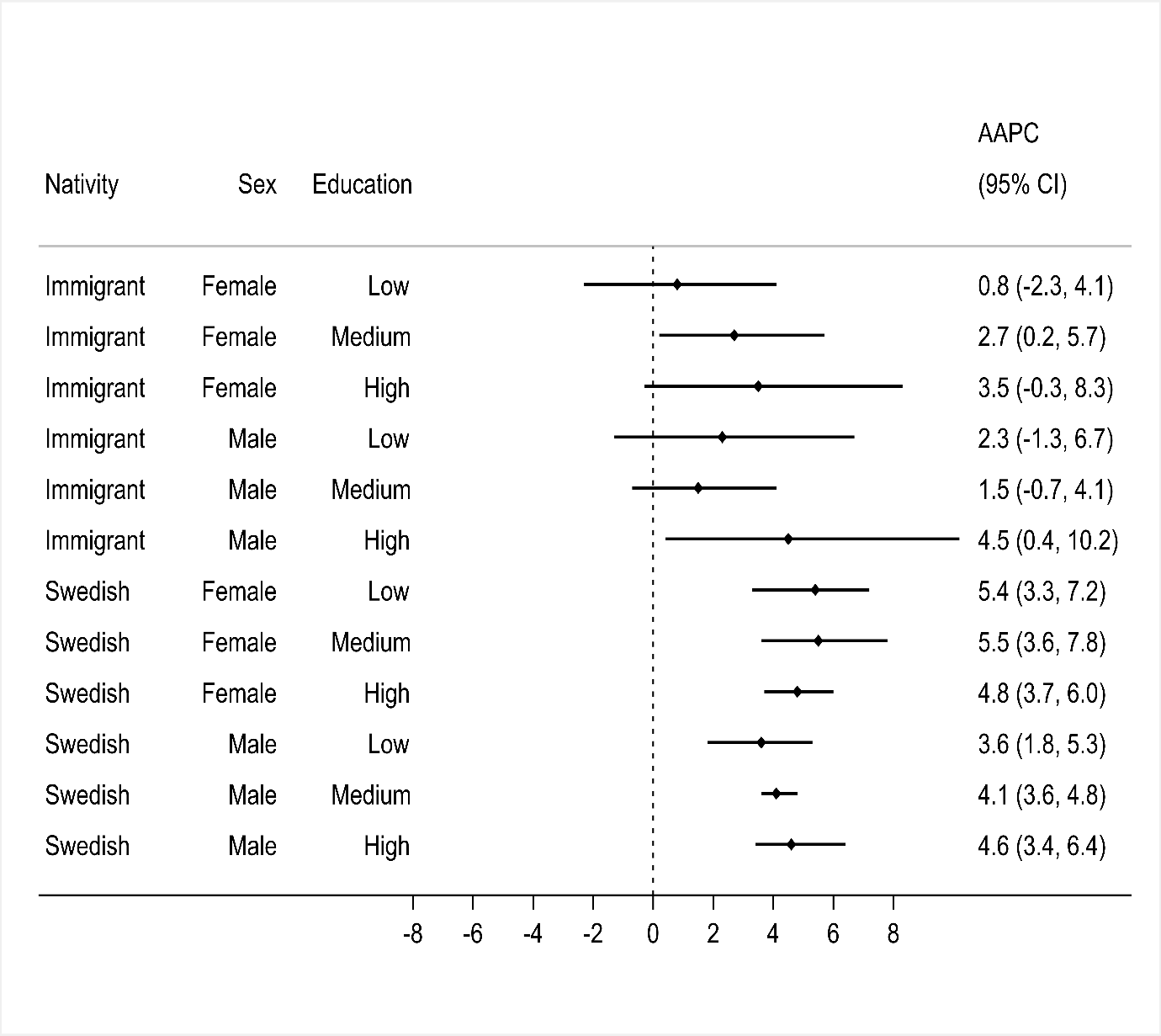
**

**Figure A7. Temporal trends in age-standardized hip osteoarthritis incidence rates across sociodemographic strata. Symbols display observed values and lines display modelled values using joinpoint regression.**


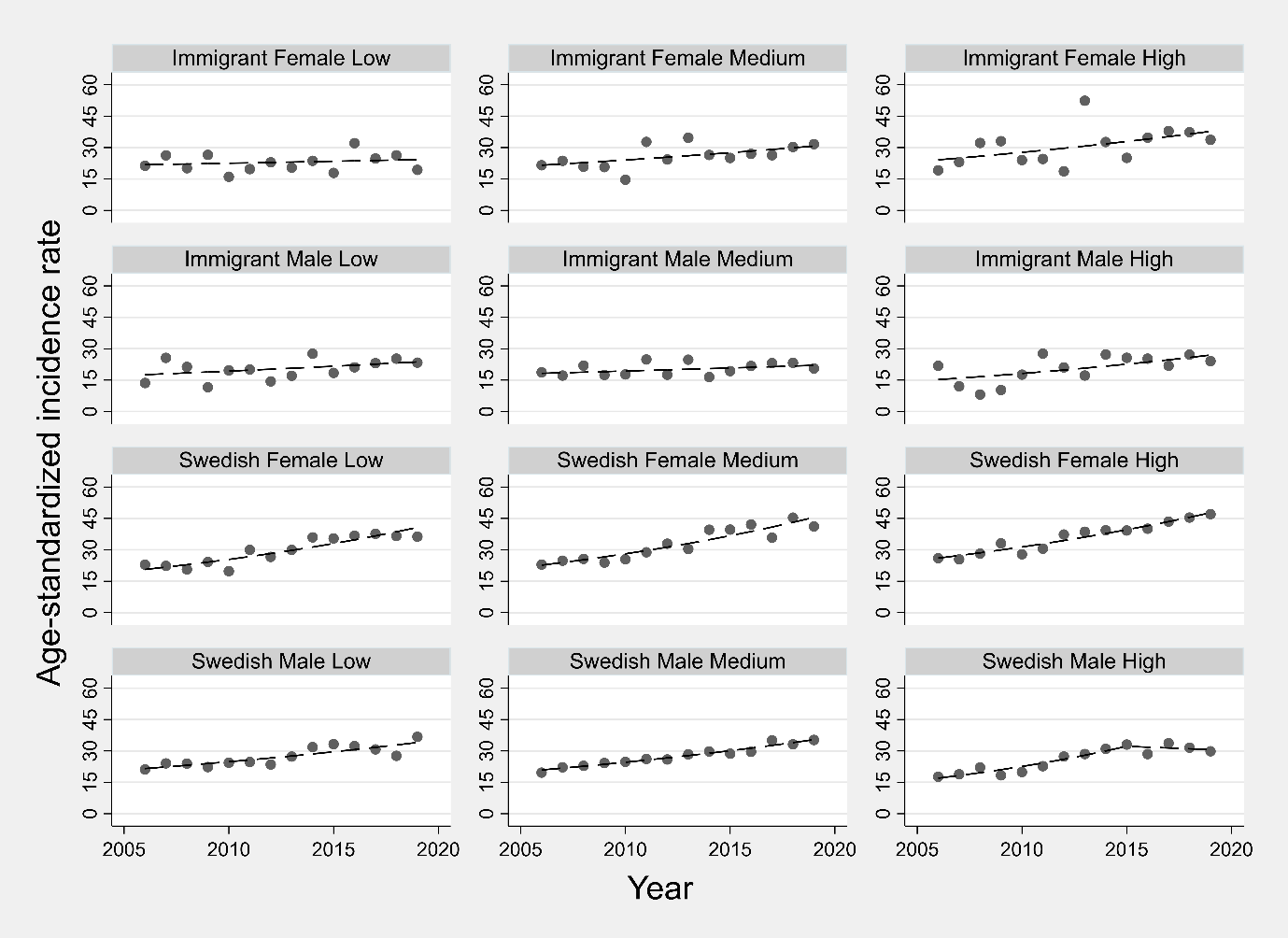


**Figure A8. Change (95% CI) in the weighted median age-at-diagnosis of knee and hip osteoarthritis (OA) between 2006-2008 and 2017-2019 across sociodemographic strata.**

**
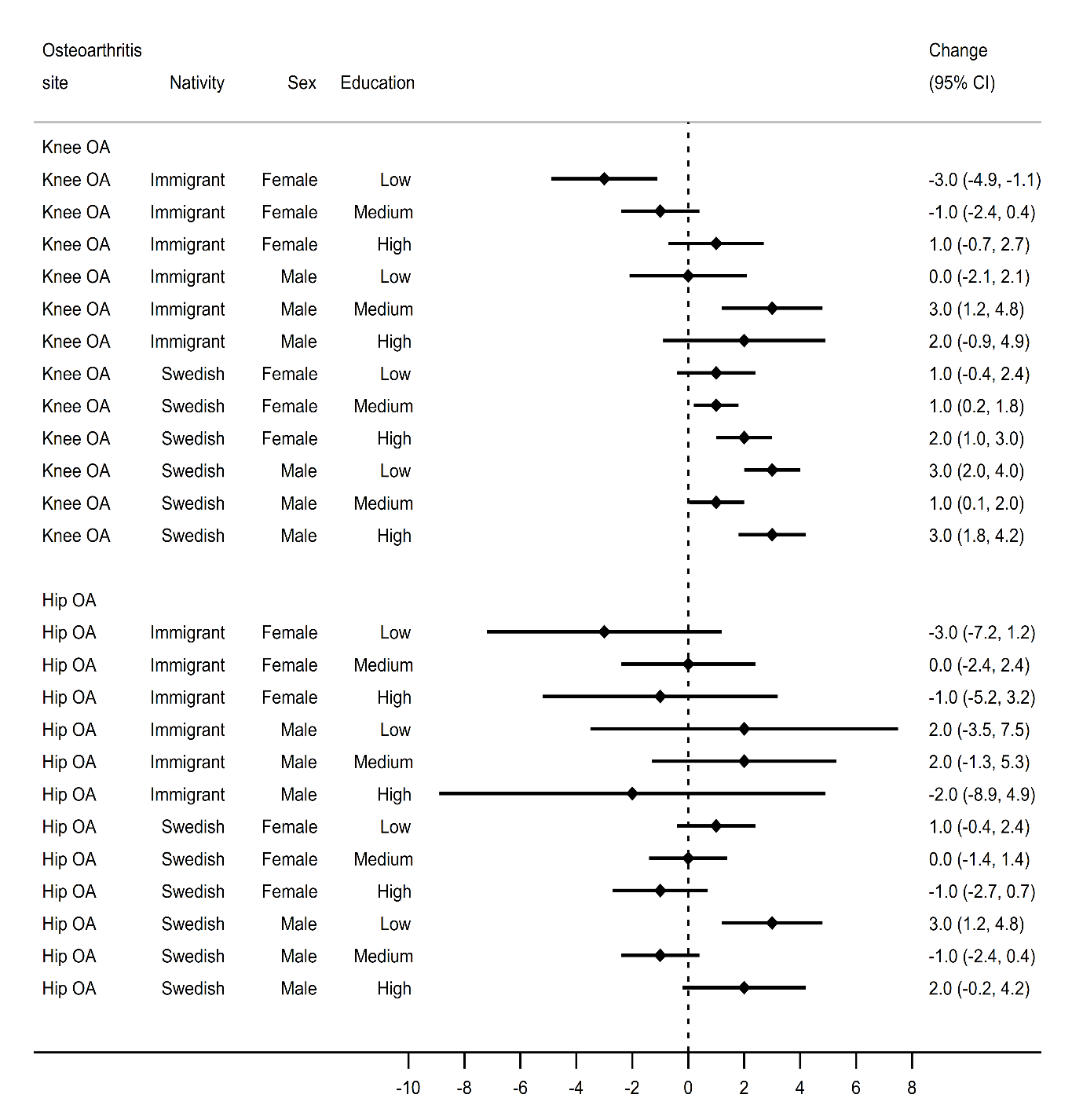
**

**Figure A9. Average annual percent change (AAPC) in age-standardized doctor-diagnosed osteoarthritis incidence rates across sociodemographic strata during 2006-2019.**


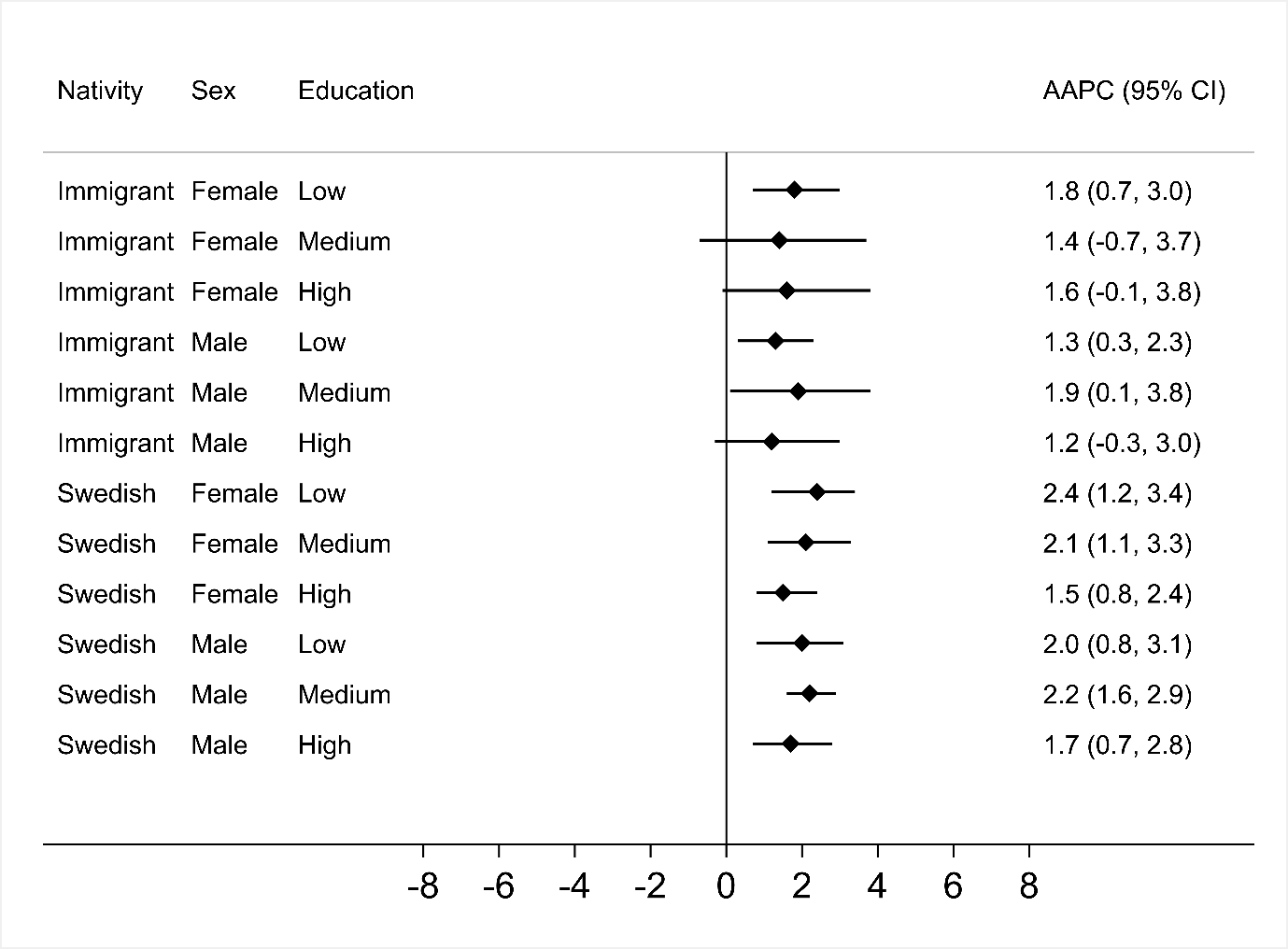


**Figure A10. Change (95% CI) in the weighted median age-at-diagnosis of doctor-diagnosed osteoarthritis between 2006-2008 and 2017-2019 across sociodemographic strata.**


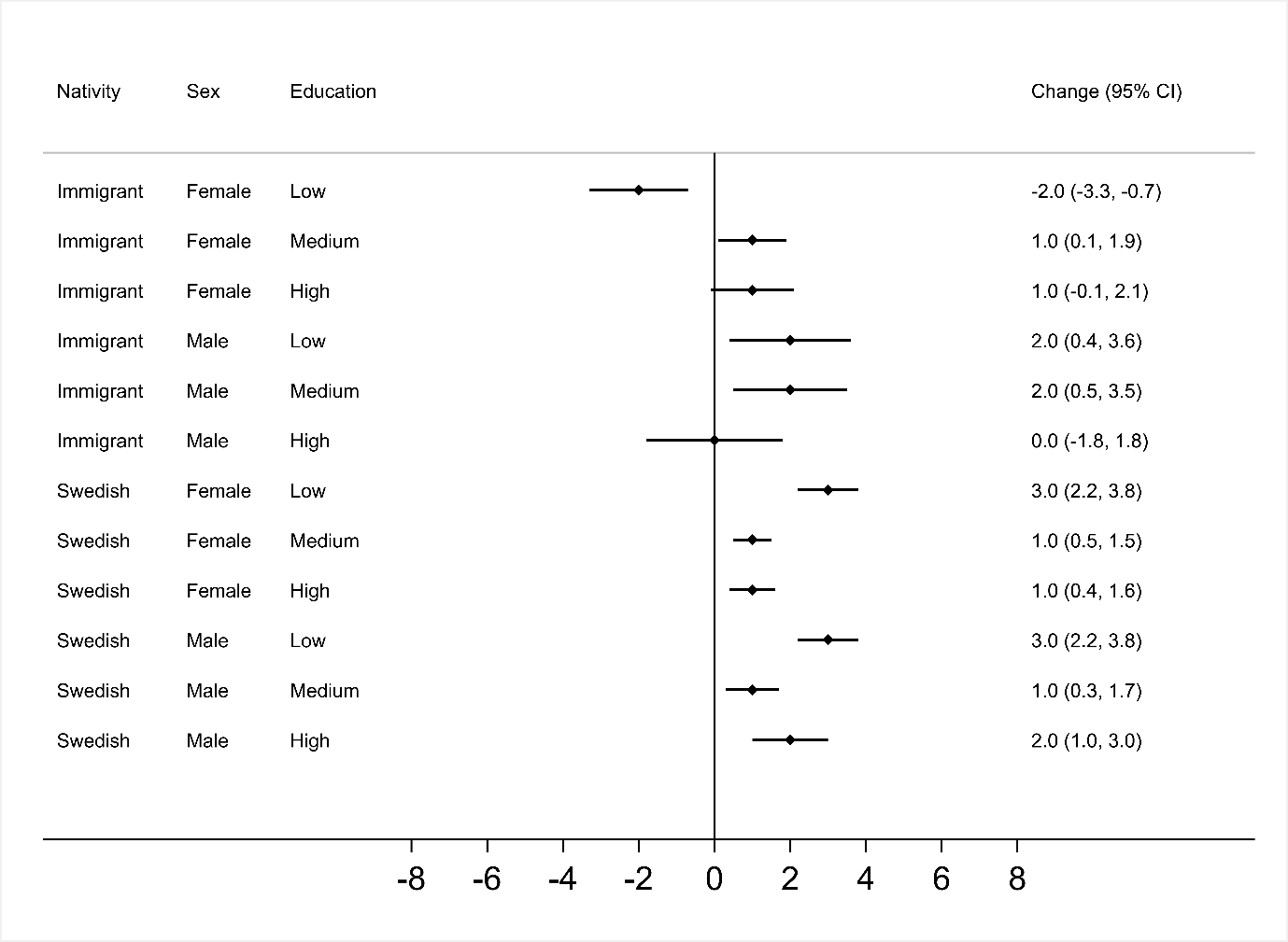

Supplement: sj-docx-1-sjp-10.1177_14034948241265427 – Supplemental material for Uncovering sociodemographic disparities in temporal trends of osteoarthritis incidence and age-at-diagnosis, 2006–2019 [file sj-docx-1-sjp-10.1177_14034948241265427.docx]
